# PtNPs/PEDOT:PSS-Modified Microelectrode Arrays for Detection of the Discharge of Head Direction Cells in the Retrosplenial Cortex of Rats under Dissociation between Visual and Vestibular Inputs

**DOI:** 10.3390/bios13050496

**Published:** 2023-04-23

**Authors:** Gucheng Yang, Yiding Wang, Zhaojie Xu, Xue Zhang, Wang Ruan, Fan Mo, Botao Lu, Penghui Fan, Yuchuan Dai, Enhui He, Yilin Song, Changyong Wang, Juntao Liu, Xinxia Cai

**Affiliations:** 1 State Key Laboratory of Transducer Technology, Aerospace Information Research Institute, Chinese Academy of Sciences, Beijing 100190, China; 2 School of Electronic, Electrical and Communication Engineering, University of Chinese Academy of Sciences, Beijing 100049, China; 3 Academy of Military Sciences, Beijing 100850, China

**Keywords:** MEA, electrophysiology, head direction cell, visual, vestibular, dissociation

## Abstract

The electrophysiological activities of head direction (HD) cells under visual and vestibular input dissociation are important to understanding the formation of the sense of direction in animals. In this paper, we fabricated a PtNPs/PEDOT:PSS-modified MEA to detect changes in the discharge of HD cells under dissociated sensory conditions. The electrode shape was customized for the retrosplenial cortex (RSC) and was conducive to the sequential detection of neurons at different depths in vivo when combined with a microdriver. The recording sites of the electrode were modified with PtNPs/PEDOT:PSS to form a three-dimensional convex structure, leading to closer contact with neurons and improving the detection performance and signal-to-noise ratio of the MEA. We designed a rotating cylindrical arena to separate the visual and vestibular information of the rats and detected the changes in the directional tuning of the HD cells in the RSC. The results showed that after visual and vestibular sensory dissociation, HD cells used visual information to establish newly discharged directions which differed from the original direction. However, with the longer time required to process inconsistent sensory information, the function of the HD system gradually degraded. After recovery, the HD cells reverted to their newly established direction rather than the original direction. The research based on our MEAs revealed how HD cells process dissociated sensory information and contributes to the study of the spatial cognitive navigation mechanism.

## 1. Introduction

The sense of direction, encoded by head direction (HD) cells in the brain, is critical for animals to recognize their surroundings and navigate effectively in space. An impaired sense of direction can cause a loss of the ability to find one’s way in locomotor environments [1,2]. When the head of an animal points in a specific direction, the corresponding HD cells discharge preferentially, with different cells having different preferred directions [3]. Head direction cells are widely distributed in multiple brain regions and interconnect to form a head direction cell system which decodes the sense of orientation in animals [4].

The retrosplenial cortex (RSC) is a crucial brain region for spatial navigation and is widely involved in memory and contextual encoding [5]. It participates in the processing of visual information via numerous connections with multiple visual areas, including the primary visual cortex [6]. In addition, the RSC has a wide distribution of angular head velocity (AHV) cells which encode angular head velocity and receive inputs from the vestibule [7]. Furthermore, studies have shown that animals with lesions in the RSC exhibited defective pathway integration in the dark [8,9]. Therefore, RSC is encoded by both visual and vestibular signals and is essential to spatial navigation behaviors involving various visual cues or self-motion.

The attractor model, an important theoretical model of HD function, suggests that when rats face a specific orientation in a visual environment, the optical input cells activate corresponding HD cells, and the angular velocity cells provide angular velocity input to encode the self-motion signal to update the HD signal [4]. In this way, HD cells can maintain a steady sense of direction even in darkness [10]. The combination of visual and vestibular inputs contributes to increasing the head direction encoding accuracy of the HD system [11,12]. However, visual and vestibular inputs can become dissociated when we take transportation and use VR devices, which differ from traditional navigation in a stable environment. The way in which HD cells process these two types of dissociated sensory information has not yet been investigated. Recent studies have started to explore the difference between self-motion and visual input to investigate the relative contributions of two sensory inputs to the spatial activity of place cells in the RSC and hippocampus [13,14] and the temporal synchrony effects of optic flow and vestibular input on heading perception [15]. Therefore, detection of the response of the HD system under visual and vestibular information dissociation remains a problem to be solved.

At present, extracellular recording of brain neurons includes neural electrodes and two-photon microscopic imaging. Two-photon imaging is a method of optical imaging to obtain neuronal discharge signals [16], which features a wide field of view and high throughput [17]. Neural electrodes are implanted into the brain of animals and directly contact with target neurons, which can obtain rich electrophysiological information such as spike discharge and local field potential [18,19], with high temporal and spatial resolution. At the same time, because of its small size and light weight, it is easier to detect electrophysiological information in the motion state. Compared to traditional wire electrodes, the MEA has good orientation and high integration, which are conducive to accurate implantation in the target brain area and the detection of high-density neurons with less implantation damage. Furthermore, the distribution of electrode sites can be customized according to the detection task, and a relatively fixed spatial distribution can be maintained during the implantation process to maximize the detection effect. In addition, MEAs prepared using MEMS technology have good uniformity and their planar site can be modified with nanomaterials to improve detection performance [20]. Indeed, Pt nanoparticles (PtNPs) have the advantages of high surface area and high conductivity, which can significantly enhance the ability to detect weak signals [21]. As a conductive polymer, poly(3,4-ethylene dioxythiophene) (PEDOT) has been verified as having a low Young’s modulus and good biocompatibility [19], and has been widely used in the biomedical field. The PtNPs and PEDOT are electrodeposited on the microelectrode, which helps to reduce the impedance of the microelectrode and improve long-term detection stability.

In our study, an MEA suitable for microdriver stepping detection was designed to locate HD cells. PtNPs and PEDOT were electrodeposited on the MEA to protrude from the electrode sites and improve the detection performance and biocompatibility. Because of the sensitivity of HD cells to directional changes, we rotated a cylindrical arena to separate the visual and vestibular signals of the rats in the directional dimension. We measured the directionality of HD cells in the RSC at the three stages of before, during, and after the separation of the visual and vestibular input of the rats. We found that within a short time, visual information had a more substantial effect on HD cells under sensory dissociation than vestibular input. Furthermore, with prolonged sensory dissociation, the function of the HD cells was gradually impaired.

## 2. Materials and Methods

### 2.1. Design and Fabrication of the MEA

**Figure 1:**
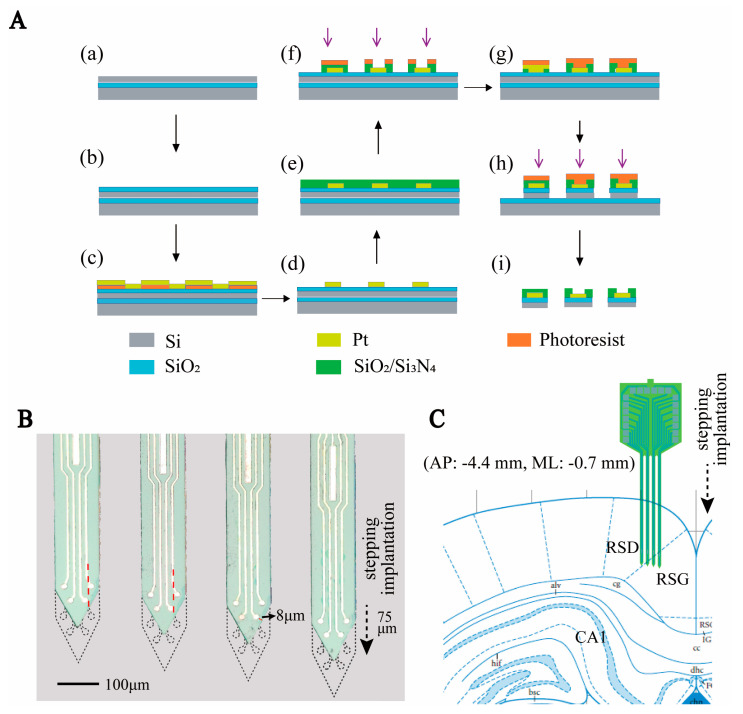
Design and fabrication of MEA. (**A**) Schematic diagram of MEMS technology for MEA fabrication. (**a**–**d**) Steps to create the conductor layer. (**e**,**f**) Steps of creating openings in the insulation layer. (**g**–**i**) Steps of silicon needle electrode release. (**B**) Micrograph of the electrode as well as the schematic diagram of the electrode stepping implantation. Where the black dashed line indicates the outline of the electrode after step implantation, the arrow indicates the direction of the step, and the red dashed line indicates the dividing line of the advance trajectory of the recording site. (**C**) Diagram of the location of the electrodes implanted in the brain area.

In this work, we designed an MEA to detect HD cells in the RSC. It consisted of four 4 mm long needles with a 110 × 26 μm^2^ (width × thickness) cross-section and a tip angle of 54°. Each needle contained four recording sites (diameter = 14 μm) and a reference site (14 μm × 146 μm). Driven by a microdriver, the MEA was lowered by 75 μm every day to monitor deeper cells. In order to monitor more cells, we designed the arrangement of electrode sites so that none of the forward tracks of the recording sites overlapped (Figure 1B). In addition, the recording sites were close to the edge (8 μm) so that, after stepping, they exceeded the original outline range of the electrode (Figure 1B). Studies have shown that approximately 12 h after MEA insertion, microglia begin moving toward the implanted MEA. After 24 h, they can surround the MEA [22]. By designing the distance between the electrode sites and the edge and using stepping, our electrode sites could be reimplanted into an area with fewer microglia which contributes to improving the electrode recording effect. In summary, we designed the lateral distribution of electrode sites and their distance to the edge of the silicon needles and used microdriver stepping electrodes to improve the detection effect of HD cells.

The MEA was fabricated in a clean room using microelectromechanical systems (MEMS) technology, as previously reported [23]. Three photo masks were designed, including conductive, insulating, and MEA probe shape layers. (1) An SOI wafer was thermally oxidized and spun using photoresist AZ5214. Then, we used an initial photolithography process to define the shape of the conductive layer. The microelectrode areas were patterned selectively by sputtered Ti/Pt (30 nm/250 nm), followed by the lift-off process (Figure 1A(a–d)). (2) A SiO_2_/Si_3_N_4_ (300 nm/500 nm) insulation layer was deposited on the conductive layer via plasma-enhanced chemical vapor deposition (PECVD, 300 °C). The insulating layer pattern was defined using the second photolithography process. Then, it was etched in a CHF_3_ environment to expose the recording sites and bonding pads (Figure 1A(e–f)). (3) The MEA probe shape layer pattern was transferred onto the wafer. We selectively deep-etched the silicon layer to form the electrode shape. Finally, the MEA was released using wet etching to remove the backer silicon (Figure 1A(g–i)). The completed MEA is shown in Figure 1B.

### 2.2. PtNPs/PEDOT:PSS Modification Method

To detect electrophysiological signals with a high signal-to-noise ratio and long time detection, the Pt nanoparticles (PtNPs) and PEDOT:PSS were electrochemically deposited on the MEAs successively. We changed the electroplating parameters to increase the protrusion of the recording sites and improve biocompatibility. Firstly, 48 mM H_2_PtCl_6_ and 4.2 mM Pb(CH_3_COO)_2_ were 1:1 mixed to obtain the PtNPs solution, and the EDOT solution was obtained by adding 20 mM EDOT to 0.1 M PSS solution and mixed ultrasonically for 30 min to ensure uniform dispersion. Next, the MEAs were electrodeposited (CA, −1.1 V, 60 s) in Pt solution to form PtNPs substrates with a protruding structure. Then, we electroplated a thick PEDOT layer in EDOT solution with parameters CV, 0~1.05 V, rate: 100 mV/s, 20 cycles to further improve the convexity and biocompatibility of the electrode sites. All the electrochemical deposition processes were carried out using electrochemical instruments (Gamry Instruments, Warminster, PA, USA).

### 2.3. Subjects and Surgery

Three male Sprague Dawley (SD) rats weighing 250 g were selected for the experiments. The rats were 3 to 6 months old and were individually housed under a 12 h light–dark schedule. Their food was slightly restricted to reduce their weight to 90% of normal free-feeding weight. All experiments were conducted with the permission of the Beijing Association on Laboratory Animal Care and approved by the Institutional Animal Care and Use Committee at the Aerospace Information Research Institute, Chinese Academy of Science (AIRCAS).

Before surgery, a self-made microdriver shuttle plate was mounted on the MEAs, enabling the MEAs to be lowered in order to detect deeper brain regions (Figure S3B). Under 1–2% isoflurane anesthesia, the rats were fixed on a stereotaxic device. We cleaned the tissue and exposed the skull, and then implanted six stainless steel screws into the skull to serve as ground electrodes. Then, we opened the skull (AP: −4.44 mm, ML: −0.7 mm) and slowly implanted the MEAs in the RSC at 0.7 mm. After implantation, the shell of the microdriver was fastened to the skull using dental adhesive, and the shuttle plate was protected with Vaseline to ensure mobility. After surgery, the rats were allowed one week to recover before the experiment commenced.

After the test, the electrode was removed, and another MEA with red fluorescent dye DiI (Beyotime, Shanghai, China) was replanted at the same position. After half an hour, the rats were anaesthetized and sequentially infused with saline and 4% paraformaldehyde (PFA) throughout the body to fix the brain tissue. Afterwards, the brain was taken and dehydrated in the sucrose solution (20% sucrose in PB 0.1 M, 30% sucrose in PB 0.1 M). Finally, the brain was sliced into 50-micron slices using a Rotary Microtome Cryostat to obtain the implantation traces of the MEA (Figure S15).

### 2.4. Apparatus

The recording environment was a cylindrical arena made of semilucent milky white acrylic (diameter: 80 cm, height: 80 cm), separated from the rest of the room by a cylindrical curtain with a diameter of 120 cm. The curtain had the same center as the arena. A black cue card (42 cm wide × 41 cm high) was attached to the inside of the wall. Opposite the cue card, a triangular LED strip (color temperature: 6000 K, powered by USB) was fixed on the outside of the wall, and its light could illuminate the inside of the arena through the wall as another visual cue. A motor was placed under the cylindrical arena and this could rotate the arena at a speed of 1.4 r/min.

### 2.5. Recording Setup

The electrical activity and behavioral data of the rats were captured simultaneously by the recording. Sixteen-channel headstage and recording cables were attached to the MEA connector. The electrical signals were amplified and stored by a neuron data recording system (AIRCAS-128, China) at 30 kHz. Highpass (>250 Hz) and lowpass (0–250 Hz) filters were used to separate neural spikes and LFPs, respectively. Two LEDs (one red and one green, spaced 6 cm apart) fixed to the headstage assembly provided the behavioral information, including the location and direction of the rat’s head. An overhead camera recorded the LEDs at 30 frames per second. Further behavioral information processing was performed using behavioral processing software (EthoVision XT16, Noldus, Beijing, China).

### 2.6. Behavioral Training and Testing Protocol

Rats were pretrained for two weeks before surgery to familiarize them with the recording environment. They were trained to forage for millet that was randomly scattered around the arena once every three minutes. For the first ten days, the rats were trained for 20 min per day in the stable arena. For the next four days, the arena was stable for 15 min and rotated for 15 min to help the rats adapt to the rotating environment. When the arena was spun, the visual and vestibular inputs of the rats dissociated so that the rats observed that they remained stationary in relation to their surroundings, but in the vestibular perception, they felt they were rotating in relation to the ground. Then, the MEA implantation surgery was performed, and the rats were allowed a week to recover.

After the rats had recovered from surgery, the electrophysiological recording sessions were initiated. Before and after recording, the cue cards and the recording arena were wiped with 75% ethanol to scramble olfactory signals. The roughness of the cylinder base was uniform, and, before recording every day, the whiskers of the rats were trimmed to within 1–2 mm above the skin surface using blunt surgical scissors. The recording sessions consisted of three groups of Control–Rotating–Recovery trials (Figure S1d–f), with each trial lasting 10 min. In Control, the arena was stable, and rats ran freely in the open field. We recorded neural activity in the RSC under these normal conditions. During Rotating, we rotated the arena to separate the visual and vestibular information of the rats, and recorded changes in the neural activity of the brain under the navigation of visual and vestibular separation. Then, in Recovery, the area was again stable, and we recorded the neural activity of the rats in their recovered state. Two hours before the daily tests, the microdriver was used to drive the MEA down in steps of 75 μm.

### 2.7. Identification and Analysis of HD Cells

The relative orientation of the green and red LEDs was used to estimate the direction of the rats’ heads. The HD tuning curve was used to describe the distribution of neuronal firing rate with direction in each bin of 10 degrees, which was measured using the following formula:

(1) $$firerate_{i}=Num\_spike_{i}/Time_{i}$$

where i=1, 2,…, 36, $Num\_spike_{i}$ was the total number of spike trains in one bin, and $Time_{i}$ was the total amount of time that the head was pointed in that bin. Then, the binned tuning curve was smoothed with a Gaussian kernel (standard deviation of 30°). The peak firing rate was defined as the rate in the bin with the highest rate, and the peak angle was defined as the angle of this bin.

Directional specificity was evaluated using Rayleigh vectors. It was determined by calculating the mean vector length from the circular distributed firing rates. Cells were considered to be head direction cells if the mean vector of the data recorded in Control was longer than the 95th percentile of the mean vector lengths in the distribution generated from the shuffled data. The value of the 95th percentile was 0.26.

To better describe the orientation specificity of HD cell tuning curves. We proposed the following formula to quantify it:

(2) Relative−Peak FR=peak firing rate/Ave

where peak firing rate was defined as the highest rate in tuning curve, and Ave was defined as the average firing rate in all directions.

## 3. Results

### 3.1. Characteristics of PtNPs/PEDOT:PSS Nanocomposites

**Figure 2:**
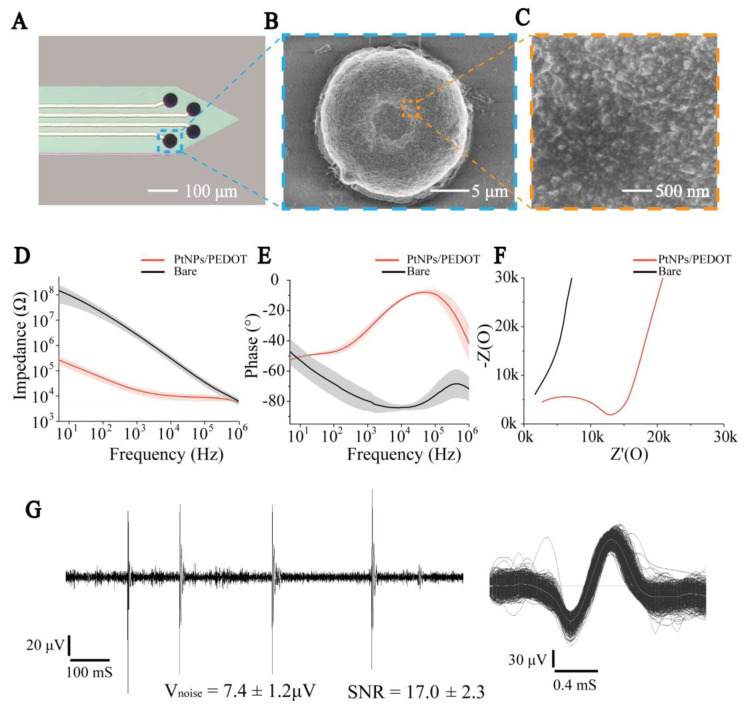
PtNPs/PEDOT:PSS nanomaterial modification and electrical performance characterization. (**A**) Micrograph of electrode sites modified by nanomaterials. (**B**) SEM image of modified nanomaterial MEA in electrode site. (**C**) SEM image of PtNPs/PEDOT:PSS. (**D**) Impedance curves of bare and modified MEAs. (**E**) Phase curves of bare and modified MEAs. (**F**) Nyquist plots of the bare and modified MEAs. (**G**) **Left**: The recorded electrophysiological signal processed by a 250 Hz high-pass filter. **Right**: Superimposed spikes isolated from the recording traces.

As shown in Figure 2A, the PtNPs/PEDOT:PSS was successfully deposited onto the surface of the microelectrodes. The SEM images showed that the PEDOT was uniformly deposited on the PtNPs, and the recording sites exhibited prominent three-dimensional structures which further increased the surface area of the microelectrode sites (Figure 2B). The three-dimensional convex structure formed by PtNPs/PEDOT:PSS is conducive to improved attachment of the recording site to the tissue and an increased signal-to-noise ratio (n = 5) (Figure S3). The PtNPs provided a rougher surface which facilitated the conductivity of the electrode sites. PEDOT electrodeposition on PtNPs effectively increased the surface area (Figure 2C), reduced the Young’s modulus of the electrode site, and improved biocompatibility [24,25].

To characterize the electrical conductivity of PtNPs/PEDOT:PSS, we compared the resistance and phase delay of bare and PtNPs/PEDOT:PSS-modified MEAs in the frequency range of 10 Hz to 100 kHz (Figure 2D,E). At 1 kHz frequency (the predominant frequency of brain electrical activity), the impedance of the MEAs significantly declined from 2682 ± 895 kΩ (bare) to 17.2 ± 5.5 kΩ (PtNPs/PEDOT:PSS) (n = 8, *** *p* < 0.001). The phase delay increased from −79.68 ± 6.74° (bare) to −32.08 ± 2.71° (PtNPs/PEDOT:PSS) (n = 8, *** *p* < 0.001), which indicated that the signal delay detected by the modified electrode was significantly reduced. In addition, Nyquist plots of the impedance spectra of the microelectrodes before and after modification indicate that PtNPs/PEDOT:PSS significantly improved the diffusion properties of the microelectrodes (Figure 2F). Then, our MEAs were tested in vivo and 250 Hz high-pass filtering was performed on the recorded electrophysiological signals. The results showed that the MEA had a low noise baseline and a high signal-to-noise ratio (n = 5) (Figure 2G and Figure S4).

### 3.2. Identification of HD and Non-HD Cells

**Figure 3:**
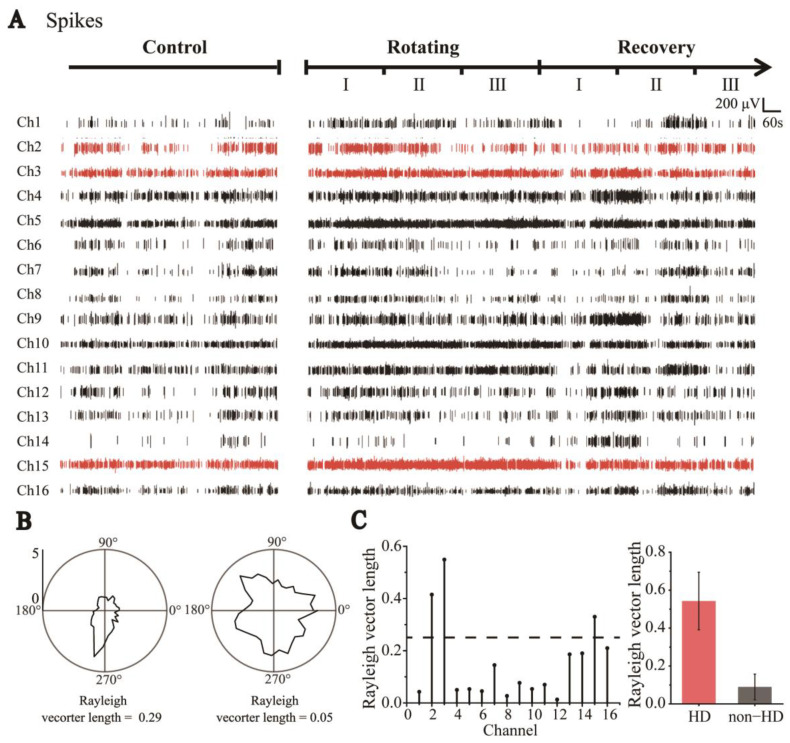
Classification of HD cells and non-HD cells. (**A**) Sixteen-channel spike signals were detected by MEAs throughout the trials, including Control, Rotating, and Recovery (Red indicates HD cell signals and black indicates Non−HD cell signals). (**B**) Polar graph of cell firing rate distributed with direction (tuning curve). Neurons whose tuning curve has an apparent peak in one direction and Rayleigh vector length greater than 0.26 are HD cells (**left**); otherwise, they are classified as non-HD cells (**right**). (**C**) The Rayleigh vector values of the cells detected in each channel (**left**). Statistical values of Rayleigh vector lengths of all detected HD and non-HD cells (HD: n = 4; non-HD: n = 20, **right**).

Neural spikes in the RSC were recorded while the rats explored the stable environment (Control), during visual–vestibular input dissociation (Rotating), and once more when stable (Recovery) in sequence (Figure 3A). For further analysis, the Rotating and Recovery stages were divided into three stages: I, II, and III;. According to the detection and classification criteria for single neuron spike firing [26,27], 24 cells were detected. As shown in Figure S6, we recorded neuronal firing with different waveforms. Because electrode sites detect different parts of the neuron in different recordings, it is difficult to distinguish HD and non-HD cells by the waveforms (Figure S7). We selected the neuronal discharges recorded in the Control stage to distinguish hypothetical HD cells from non-HD cells. The directional tuning curve (10° resolution) for each cell was computed by dividing the total number of spikes detected when the head of the rat was pointed in a particular direction by the total time spent in that orientation. A pair of tuning curves of HD and non-HD cells are shown in Figure 3B. The HD score, defined as the average Rayleigh vector length of the tuning curve, was usually standardized to measure HD specificity (see Methods). Based on previous studies [28], cells with a vector length above a threshold of 0.26 were considered to be HD cells, as shown at the bottom of Figure 3B; otherwise, they were defined as non-HD cells. Using this method, we screened four groups of HD cells and 20 groups of non-HD cells (Figure 3C).

### 3.3. Degraded Directional Tuning of Head-Direction Cells Induced by Sensory Dissociation

**Figure 4:**
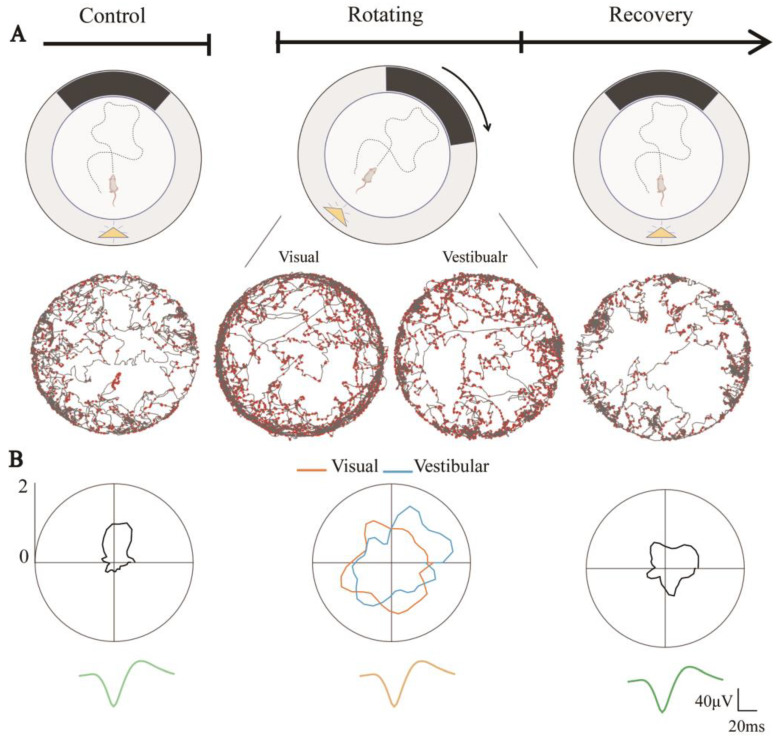
Directional tuning of HD cells degraded due to visual and vestibular input dissociation. (**A**) Schematic diagram of sensory dissociated trials including Control, Rotating, and Recovery, where the yellow triangle represents the light source and the black band represents the visual marker (**top**) and example of an HD cell in the corresponding phase, showing spike position dots (red) in trajectory maps (gray) under the rat’s visual and vestibular reference frames (**bottom**). (**B**) Directional tuning curves of a head direction cell in three trails (**top**), where orange lines indicate its tuning curves in the visual reference frame and blue lines in the vestibular reference frame. The average spike waveforms of the neurons are consistent in the three phases (**bottom**).

Two rats were trained to explore a circular arena surrounded by an 80 cm high wall. To investigate the effect on HD cells of the separation of visual and vestibular inputs, we rotated the arena continuously at 1.4 r/min to separate the rats’ visual and vestibular information (Figure 4A). Because the roles of the two types of information were unclear, we analyzed the directionality of HD cells under the visual (i.e., relative to the arena) and vestibular (i.e., relative to the ground) frames of reference of the rats separately. The rats maintained their speed during the Control, Rotating, and Recovery trials (n = 4, *p* > 0.05) (Figure S8), which indicated that the rotation of the arena had little effect on their movement.

Throughout the experiment, the neuronal discharge waveform remained constant. The directional tuning of HD cells during Rotating in both the visual and vestibular reference frames was significantly degraded compared to Control (Figure 4B). This might be due to the dissociation of visual and vestibular inputs. From Figure 4B, we can also observe that the tuning curves in Recovery showed weak directional tuning. Whether this was due to the simple weakening of the directionality of the HD cells or because the discharge of HD cells changed from nondirectional to directional in Recovery, resulting in a poor directional firing in the overall stage, is worthy of further investigation.

### 3.4. Directional Tuning of HD Cells Is Dominated by Visual Rather than Vestibular Input during Short-Time Dissociated Navigation

To study more detailed changes in the directionalities of HD cells, Rotating and Recovery were each divided into three equal sequential segments, referred to as RotatingI, RotatingII, RotatingIII, RecoveryI, RecoveryII, and RecoveryIII, as shown in Figure 3A.

**Figure 5:**
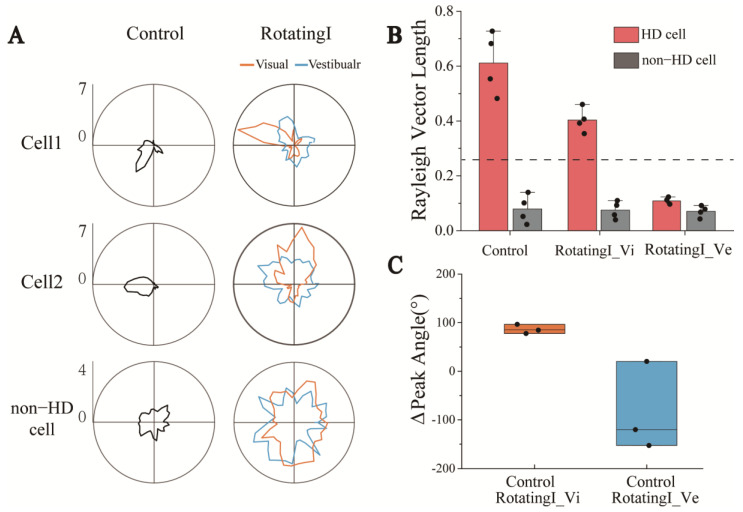
HD cells maintained directional tuning in the visual reference frames under short-term visual and vestibular input dissociation. (**A**) Tuning curves of two HD cells recorded simultaneously with one non-cell under the visual and vestibular reference frames in RotatingI and Control. (**B**) Statistics of HD cells’ Rayleigh vector length in visual and vestibular reference frames revealed that HD cells maintained their directional specificity in the optical reference frame rather than in the vestibular. The dashed line indicates the threshold of Rayleigh vector length, above which the HD cells were identified. (**C**) The Δpeak angle of three simultaneously recorded HD cells from Control to RotatingI_Vi (orange) and Control to RotatingI_Ve (blue) revealed that the shift of the peak angle under the visual reference frame is more coherent than in vestibular.

In order to study the change process of directional discharge of HD cells in detail, we first analyzed the changes in the HD cells during a short period from a stationary environment to a rotating environment. In Rotating, the visual and vestibular inputs of the rats were dissociated because of the rotation of the arena. In Figure 5A, we present the examples of two simultaneously recorded HD cells to show the tuning curves from Control to RotatingI in the visual and vestibular reference frames, with changes in the non-HD cells as a control group. As Figure 5A shows, the tuning curve of the HD cells in RotatingI showed an apparent directional preference in the visual frame of reference (orange), whereas no directional tuning was exhibited in the vestibular frame of reference (blue). A reasonable explanation for this is that HD cells were strongly anchored to the visible markers in the arena, and their preferred direction changed with the rotation. To further verify this, we computed the HD score under both reference frames to quantify the directional tuning. As shown in Figure 5B, the vector length in the visual frame exceeded the confidence value (0.43 > 0.26, n = 4), but was below it (0.12 < 0.26, n = 4) in the vestibular frame of reference. In addition, non-HD cells showed no apparent directional discharge characteristics in either frame of reference. Moreover, the Relative-Peak FR in RotatingI_Vi is significantly greater than that in RotatingI_Ve (Figure S13). It further indicated that the directional tuning of HD cells in the visual reference frame was stronger than that in the vestibular reference frame. We therefore suggest that the function of the HD cells remained, and they exhibited directionality in the visual frame of reference.

Next, to further investigate the stability of the HD system, we analyzed the peak direction change of three HD cells recorded simultaneously in one experiment. We found that the preferred directions drifted coherently in the visual frame of refence (Figure S9A) but were scrambled in another frame (Figure S9B). In addition, the changes of direction in the optical frame were more concentrated (about 100°), whereas those in the vestibular frame were more scattered (Figure 5C). The attractor model, a well-known theoretical foundation of HD function, says that a group of HD cells with similar firing directions activate one another while inhibiting those with more diverse preferred directions, which allows the difference between the preferred directions of the HD cells to be preserved. Therefore, the drift of individual HD cells will be coherent, that is, by the same amount and in the same direction [29,30]. This theory is consistent with the coherent drift of the preferred direction of individual HD cells from Control to RotatingI stages under the visual frame of reference observed in this study, which further indicates that the HD system is functional in RotatingI.

### 3.5. Long-Term Dissociation between Visual and Vestibular Perception Degrades the Directional Tuning of HD Cells

**Figure 6:**
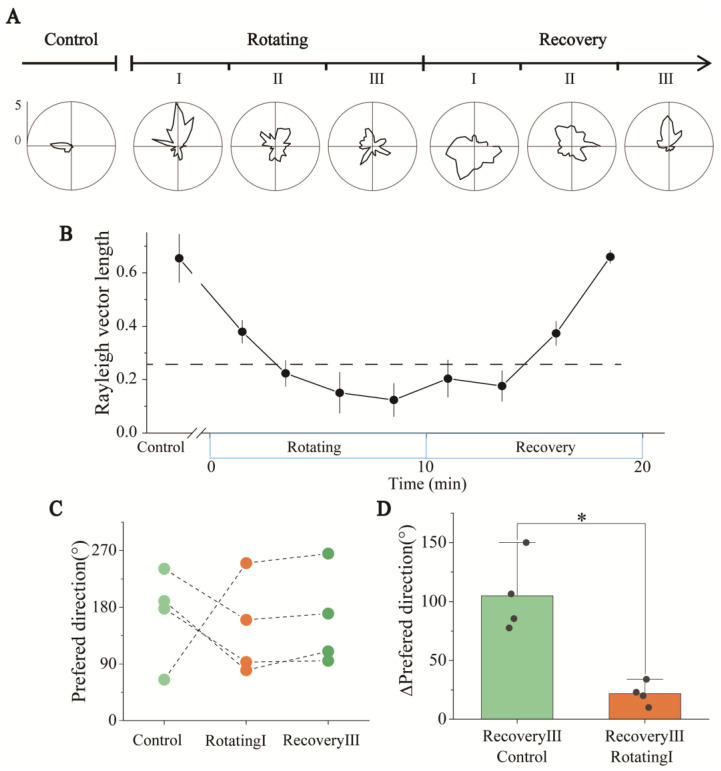
Direction tuning and statistics of HD cells in Rotating and Recovery under the visual reference frame. (**A**) Changes in the detailed tuning curve of an HD cell in Control, Rotating, and Recovery. (**B**) Statistics of Rayleigh vector lengths revealed that the directionality of HD cells was impaired over the time of sensory conflict in Rotating and gradually recovered in Recovery (n = 4). The dashed line represents the threshold of HD scores, above which a significant directivity is indicated. (**C**) Changes in the preferred directions of the three HD cells recorded simultaneously when they had directional tuning, including Control (light green), RotatingI (orange) and RecoveryIII (dark green). (**D**) ∆Preferred direction of HD cells from RecoveryIII to RotatingI (green) and RecoveryIII to Control (orange). (* *p* < 0.05, paired *t*-test, n = 4).

The detailed tuning curves of two simultaneously detected HD cells throughout the experiment are shown in Figure 6A as examples. Because the HD cells were anchored to the visual frame of reference, we only analyzed the activity of HD cells in the visual frame of reference in the following analysis. The tuning curves in the three periods of the Rotating stage showed that, from RotatingI to RotatingIII, the directionality of HD cells gradually degraded in the visual frame of reference. To better quantify the changes in directional tuning, the Rayleigh vectors in the Rotating stage were computed. The results showed the HD scores decreasing with the time of sensory dissociation (Figure 6B). Only the first three minutes showed apparent directional tuning (vector length: 0.38 ± 0.04 > 0.26, n = 4). This illustrated that the directional tuning of the HD cells gradually degraded and disappeared with the dissociation time of the visual and vestibular inputs of rats.

We then analyzed the directional changes of the HD cells after the termination of sensory dissociation. As shown in Figure 6A and Figure S10, the tuning curves of each period showed that after a period of stationary recovery, the HD cells recovered from nondirectionality (RecoveryI stage) to apparent directional tuning (RecoveryIII stage). The vector length also increased over time during the Recovery stage (Figure 6B). This meant the function of HD cells recovered gradually after the dissociation stopped.

To better understand the changes in internally referenced tuning to head-direction displacement in the experiment, we selected periods when the directional tuning was significant to analyze changes in the internally referenced tuning of rats, including Control, RotatingI, and RecoveryIII stages. The firing directions of three simultaneously recorded HD cells in these three stages are shown in Figure 6C. A directional shift was evident from the Control to RotatingI stages (Figure 6A,C), probably caused by the sudden dissociation of visual and vestibular signals. In the RecoveryIII stage, the firing direction after recovery was apparently closer to the direction in RotatingI than in Control stage (Figure 6C). Figure 6D calculates the Δpreferred direction between different periods, showing that the directional difference between the RecoveryIII and RotatingI stages was significantly smaller than that between the RecoveryIII and Control stages. To analyze the relationship between the directional tuning (“instability”) of HD cells in three periods, we calculated the Kullback–Liebler Divergence (KLD) among them to quantify the differences [31,32]. The KLD of RotatingI_Vi × RecoveryIII in the visual frame of reference was significantly lower than others (Figure S12), which means that the directional tuning in RecoveryIII and RotatingI_Vi were similar. In conclusion, the directional tuning disappeared with the time of sensory dissociation and recovered gradually after the dissociation stopped. Furthermore, the preferred direction recovered to that observed in RotatingI rather than the Control stage.

## 4. Discussion

In this study, an MEA was designed and fabricated to reliably find HD cells. The PtNPs/PEDOT:PSS-modified sites, characterized by a three-dimensional convex structure, increase the surface area and contact with tissues and effectively improve the signal-to-noise ratio and electrical performance. Three approaches were used to improve the ability to find HD cells, including the design of the lateral distribution of the electrode sites, a reduction in the distance between the recording sites and the edge of the silicon needle, and the daily use of a microdriver stepping electrode.

To explore the influence on the HD system, the visual and vestibular signals of the rats were dissociated by rotating the arena. We found that when subjected to a short period of sustained sensory dissociation, the HD cells anchored to the visual reference frame and their directional preference drifted. With prolonged and continuous dissociation of the two signals, the directional tuning of the HD cells gradually degraded. After the dissociation ended, they slowly reverted to the direction they established based on visual information rather than to the original direction.

During short periods of visual and vestibular input conflict, changes in the directionality of the HD cells were used to explore the contributions of the two signals. The inputs of both visual and vestibular sensory information play an essential role in the directional tuning of HD cells in the RSC [33,34]. In our experiment, when the two sensory inputs were dissociated for a short time, the apparent directional tuning and consistent drift of the peak directions of the HD cells in the visual frame of reference contrasted with the implicit directionality and scrambled shift of peak angles in the vestibular frame of reference (Figure 5). We suggest that the HD cells retain their directional tuning and remain anchored to the optical frame of reference during short periods of sensory dissociation. The HD cells bound more strongly to visual signals than vestibular inputs. A recent study used calcium imaging to record the place cells firing when mice watched VR virtual images to mediate their vision and vestibular senses, finding that visual signals overrode locomotion signals [13]. Using an MEA to detect the electrophysiological signals of neurons, we also observed that head direction cells were dominated by visual information input compared with vestibular perception. This result is consistent with our navigation in the internal environment of buses in daily life.

After entering the rotating environment, HD cells anchored to a new direction based on visual information (Figure 5A), which may be due to the sudden rotation of the arena forming a new environment, and the change in environmental conditions causing a drift in the anchored orientation of the HD cells.

In response to long-term and continued visual–vestibular dissociation, the directional tuning of HD cells degraded significantly. The RSC brain region receives both visual and vestibular information [33,35] and is the critical brain region integrating the two types of sensory information. At the same time, HD cells also integrate information in brain regions such as the ADN and project between HD cells in multiple brain regions [36]. In addition, visual and vestibular information play different roles in HD cell circuits in different brain regions [4]. It is possible that long-term information asynchronism increases the difficulty of information integration in the brain, which gradually causes dysfunction of the information integration function and thus leads to the degeneration of the directional tuning ability of the HD system (Figure 6A,B). This result is consistent with the poor orientation caused by car sickness or the prolonged use of VR devices.

Similar to the report from a previous experiment [37], we noticed a shift in the firing direction of HD cells in the RecoveryIII compared to Control stage. In this study, the firing direction shift occurred at the moment of transition from a stationary to a rotating environment. Throughout the experiment, the HD cells showed obvious directionality in three periods: Control, RotatingI, and RecoveryIII. In the Recovery stage, the preferred direction of the HD cells reverted to the direction newly established in the RotatingI stage (Figure 6A,C), indicating that the new direction established during the RotatingI stage was stable and the rats remembered the area environment in its rotating state. Finally, the HD cells reverted to the direction newly established in the RotatingI stage rather than the original direction in the Control stage. This phenomenon may be consistent with the example of people experiencing disorientation when suddenly getting out of a car after having been asleep during the journey. If we do not correct the directional deviation between ourselves and the external environment in time due to falling asleep or other reasons, our internal direction will be different from the actual direction of the outside world. When we get out of the car, we will feel disoriented and need to correct our sense of direction. So, in the navigation of both internal and external environments, it is necessary to constantly update the direction with visual information to maintain correct awareness of direction.

In this study, we revealed the phenomenon of orientation anchoring and degradation of HD cells under sensory dissociation. However, we need to acknowledge that this experiment also has some limitation, rotation speed is an influential factor in studies of visual and vestibular dissociation. A slowdown or increase in rotation speed might have a different effect on the behavior of HD cells. In this experiment, we revealed the behavior of HD cells under sensory inconsistency at an appropriate rate. It would be interesting and useful to further explore the effects of different rotation speeds on HD cells and to find a speed threshold that allows the HD system to deal with sensory inconsistencies below it. In addition, it would be meaningful to explore ways to enhance the HD system’s ability to deal with sensory inconsistencies in combination with electrical stimulation therapy.

## 5. Conclusions

In this study, we customized the MEA shape and site arrangement for the step detection method in order to effectively detect HD cells. Moreover, the PtNPs/PEDOT:PSS modification caused the electrode site to protrude, effectively increasing the surface area of the site and its contract to neurons and improving the detection signal-to-noise ratio. The electrophysiological results showed that, under short-term sensory dissociation, HD cells established directional tuning based on visual information. In addition, long-term sensory dissociation degraded the directional tuning of HD cells. Ultimately, the firing direction of the HD cells reverted to the direction newly established with visual information rather than to the original orientation. These findings explain how our sense of direction changes when we navigate under sensory dissociation (for example, when taking transportation or using VR).

## Data Availability

Data is contained within the article or Supplementary Material.

## Supplementary Materials

The following supporting information can be downloaded at: https://www.mdpi.com/article/10.3390/bios13050496/s1, Figure S1: Schematic diagram of the sensory dissociated rat experiment. (a) Fabrication of MEAs. (b) PtNPs/PEDOT were electrodeposited onto the MEA to enhance electronical performance of the electrodes. (c) MEAs were surgically implanted into the RSC brain region of rats. (d) Rats were freely explored in a static open field for screening HD cells. (e) The open field was rotated to separate the visual and vestibular information of the rat to detect changes in HD cell discharge. (f) The rotation of the open field was stopped and the firing changes of neurons in the RSC during the recovery phase were examined, Figure S2: The mask design and structure diagram of the 16-channel electrode used in this experiment. (A) Three mask layers of MEA, including insulation layer (top), silicon needle pattern layer (middle), conductive layer (bottom). (B) Schematic diagram of electrode sandwich structure fabricated, Figure S3: Equipment diagram of electrodes with micro-driver. (A) Structure diagram of the microdriver. The middle part is a screw and hexagonal nut (M1.4), which can drive the shuttle plate up and down by twisting the screw. (B) Assembly diagram of the MEA and micro-driver, Figure S4: Spike waveform and the noisy baseline were detected simultaneously. (A) Noisy baseline (5.25 μV) during electrophysiological recordings. (B) Neuronal firing waveform was simultaneously recorded by the channels shown in (A). Signal-to-noise ratio (S/N): 224/10.5 ≈ 21, Figure S5: Top view of the scene in which rats perform tests in the arena, Figure S6: Waveforms of all detected HD cells (n = 4, red) and Non-HD cells (n = 20, black), Figure S7: A total of 24 single-neuron discharge units were obtained from spike signals recorded from 16 channels of the MEA and were then classified into 4 HD cells and 20 Non-HD cells. (A) The parameters for calculating spike duration and symmetry index. “a” represents the amplitude of pre-peak while “b” represents that of the post-peak. Symmetry index = (b − a)/(a + b). (B) The distribution of HD and Non-HD cells with spike duration and symmetry index parameters, Figure S8: Statistical graphs of the mean motor speed of the rats in the three trials (n.s., paired *t*-test, n = 4), Figure S9: Changes of peak direction of three simultaneously recorded HD cells in the visual reference frame and vestibular reference frame from Control to RotatingI. (A) Changes of peak direction of HD cells under the visual reference frame showed that the shift of the peak direction is coherent. (B) Changes of peak direction of HD cells under the vestibular reference frame showed that the shift of the peak direction is scrambled. Figure S10. Detailed directional tuning curve changes of HD cells in the visual reference frame in Control, Rotating, and Recovery, Figure S11: Detailed directional tuning curve changes of HD cells in the vestibular reference frame in Control, Rotating, and Recovery, Figure S12: The divergence (KLD) of HD cells’ tuning curves in RecoveryIII relative to RotatingI and Control revealed that the directional tuning of HD cells is closer to in RotatingI_Vi than in Control, Figure S13: The Relative-Peak FR of RotatingI_Vi was significantly higher than that of RotatingI_Ve, indicating that HD cells showed more significant directional tuning in the visual reference frame than in the vestibular reference frame, Figure S14: The PSD of LFP in RSC has a characteristic peak in the theta band, Figure S15: Postmortem histochemistry. (A) staining trajectory of electrode implantation in brain slices. (B) Dil fluorescence staining diagram of electrode implantation position; Video S1: Behavioral experiment scene. Control: the open field was stable, in which the rats were free to explore the open field; Rotating: the open field was rotating, in which the rats’ visual information was separated from vestibular information; Recovery: the open field was returned to stable, the rat was resting in the open field.
