# Supplementary material for: PtNPs/PEDOT:PSS-Modified Microelectrode Arrays for Detection of the Discharge of Head Direction Cells in the Retrosplenial Cortex of Rats under Dissociation between Visual and Vestibular Inputs
Source: Biosensors (Basel). 2023 Apr 23;13(5):496. doi: 10.3390/bios13050496 (PMC10216047; doi:10.3390/bios13050496)
Supplement: Supplementary file 1 [file biosensors-13-00496-s001.zip › biosensors-2266646-supplementary.pdf]

biosensors
MDPI

Supporting Information

PtNPs/PEDOT:PSS-Modified Microelectrode Arrays for Detection of the Discharge of Head Direction Cells in the Retrosplenial Cortex of Rats under Dissociation between Visual and Vestibular Inputs

Gucheng Yang, Yiding Wang, Zhaojie Xu, Xue Zhang, Wang Ruan, Fan Mo, Botao Lu, Penghui Fan, Yuchuan Dai, Enhui He, Yilin Song, Changyong Wang, Juntao Liu and Xinxia Cai

Figure S1

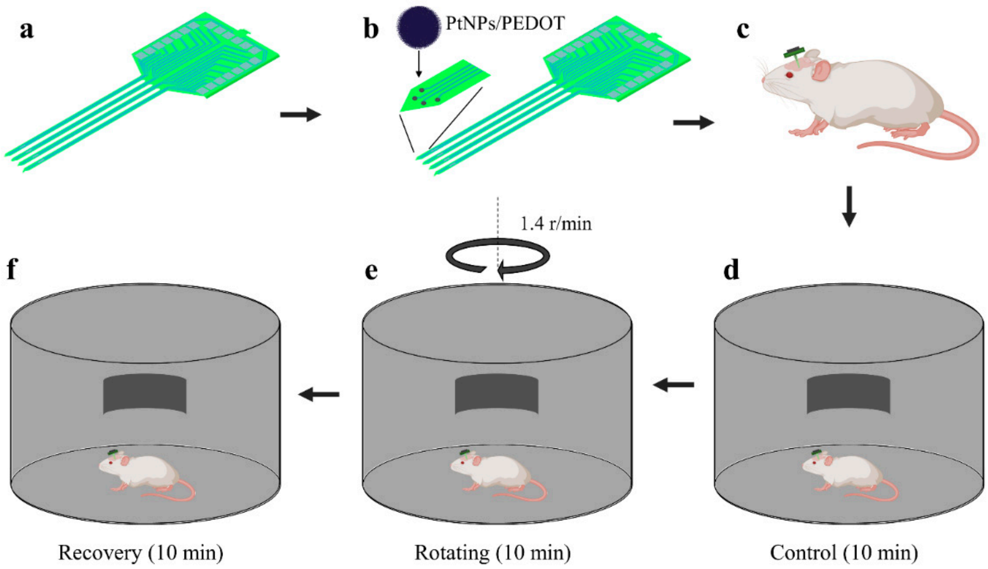

Figure S1. Schematic diagram of the sensory dissociated rat experiment. (**a**) Fabrication of MEAs. (**b**) PtNPs/PEDOT were electrodeposited onto the MEA to enhance electronical performance of the electrodes. (**c**) MEAs were surgically implanted into the RSC brain region of rats. (**d**) Rats were freely explored in a static open field for screening HD cells. (**e**) The open field was rotated to separate the visual and vestibular information of the rat to detect changes in HD cell discharge. (**f**) The rotation of the open field was stopped and the firing changes of neurons in the RSC during the recovery phase were examined.

Figure S2

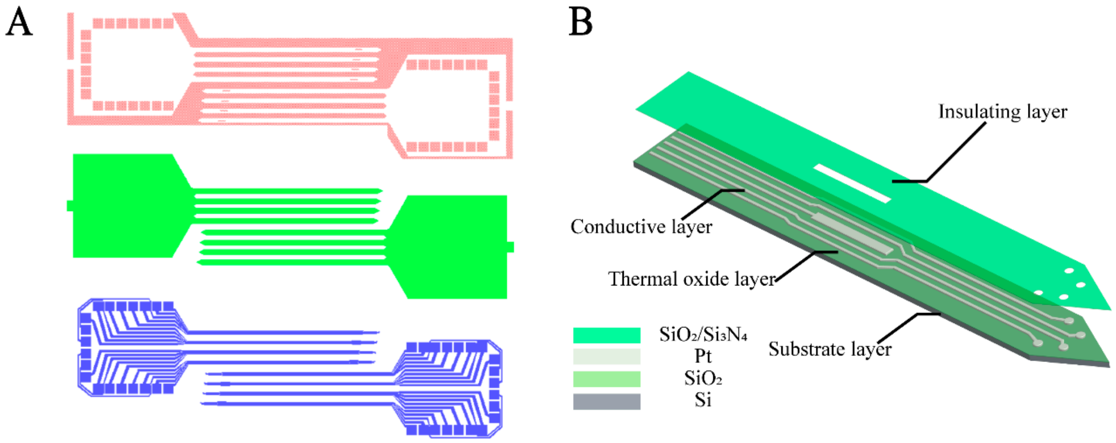

Figure S2. The mask design and structure diagram of the 16-channel electrode used in this experiment. (**A**) Three mask layers of MEA, including insulation layer (top), silicon needle pattern layer (middle), conductive layer (bottom). (**B**) Schematic diagram of electrode sandwich structure fabricated.

Figure S3

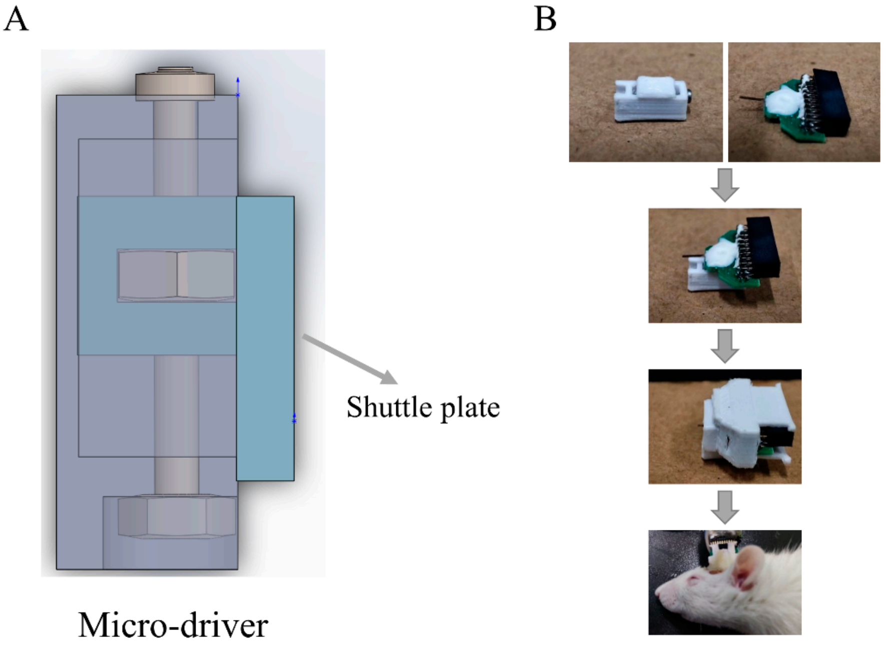

Figure S3. Equipment diagram of electrodes with micro-driver. (**A**) Structure diagram of the micro-driver. The middle part is a screw and hexagonal nut (M1.4), which can drive the shuttle plate up and down by twisting the screw. (**B**) Assembly diagram of the MEA and micro-driver.

Figure S4

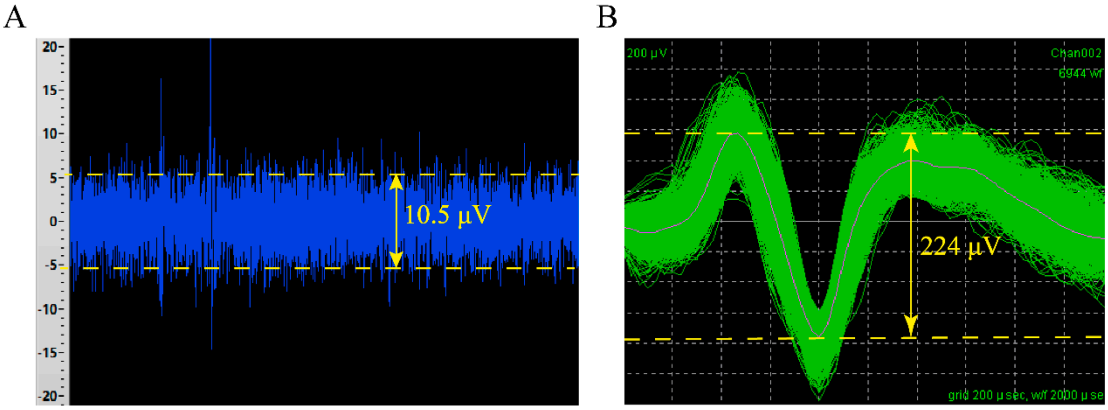

Figure S4. Spike waveform and the noisy baseline were detected simultaneously. (**A**) Noisy baseline (5.25 µV) during electrophysiological recordings. (**B**) Neuronal firing waveform was simultaneously recorded by the channels shown in (A). Signal-to-noise ratio (S/N): 224/10.5 ≈ 21.

Figure S5

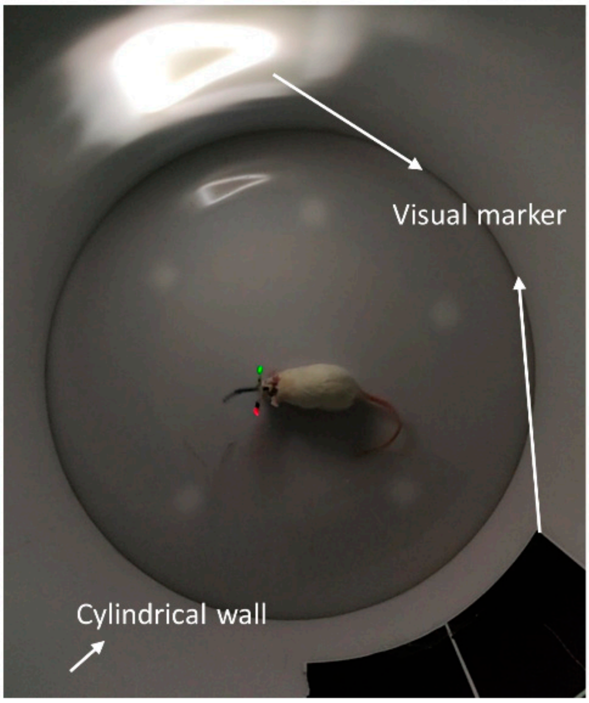

Figure S5. Top view of the scene in which rats perform tests in the arena.

Figure S6

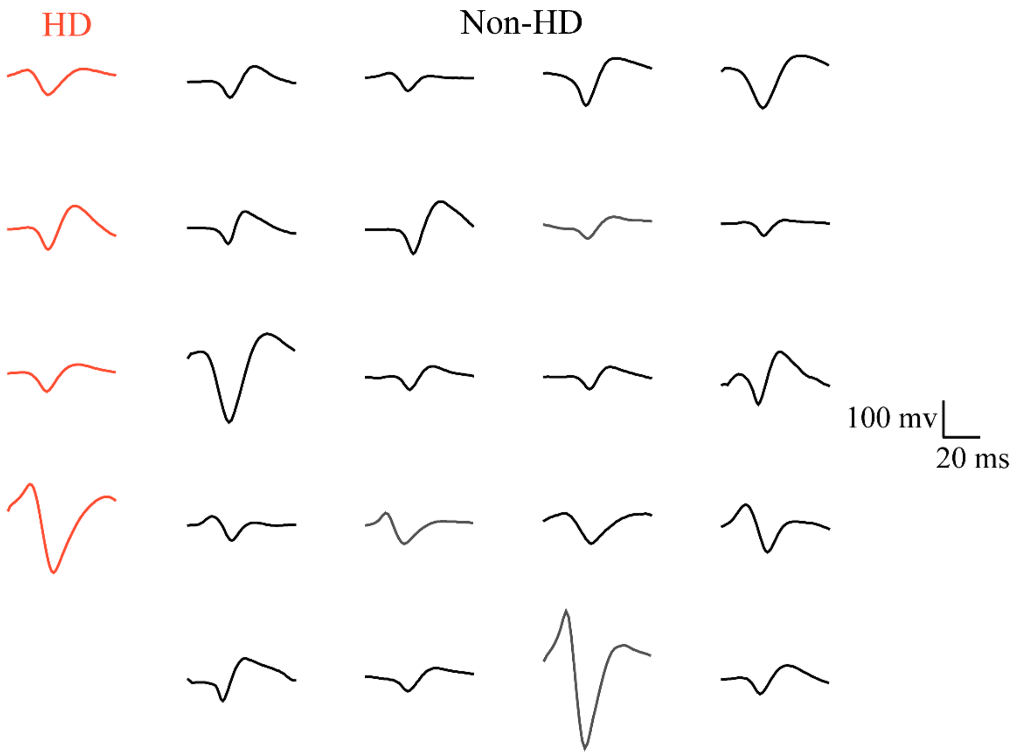

Figure S6. Waveforms of all detected HD cells (n =4, red) and Non-HD cells (n = 20, black).

Figure S7

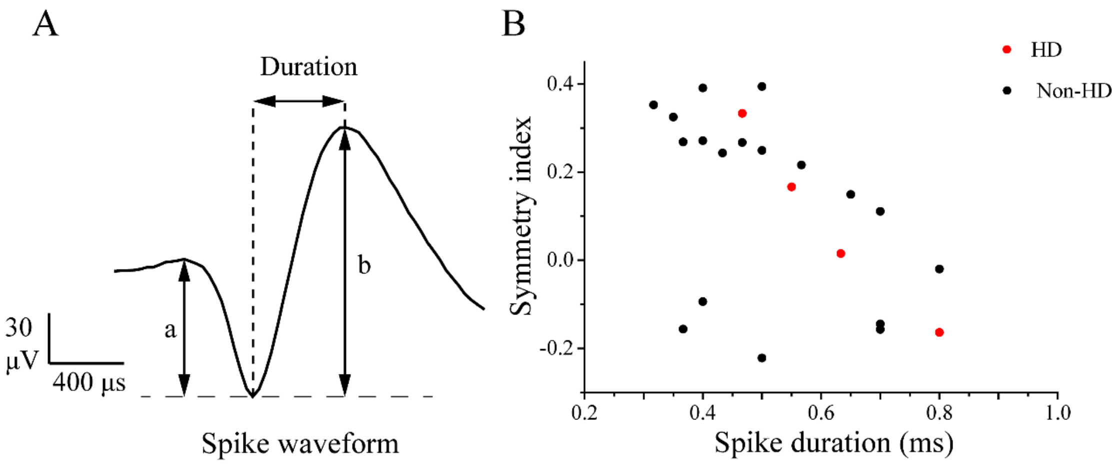

Figure S7. A total of 24 single-neuron discharge units were obtained from spike signals recorded from 16 channels of the MEA and were then classified into 4 HD cells and 20 Non-HD cells. (**A**) The parameters for calculating spike duration and symmetry index. "a" represents the amplitude of pre-peak while "b" represents that of the post-peak. Symmetry index = (b-a)/(a+b). (**B**) The distribution of HD and Non-HD cells with spike duration and symmetry index parameters.

Figure S8

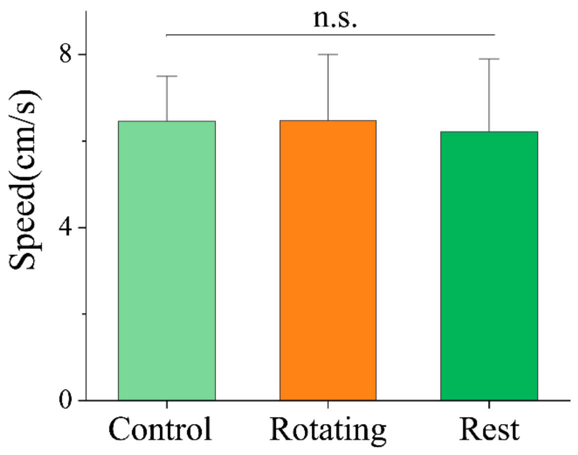

Figure S8. Statistical graphs of the mean motor speed of the rats in the three trials (n.s., paired t-test, n = 4).

Figure S9

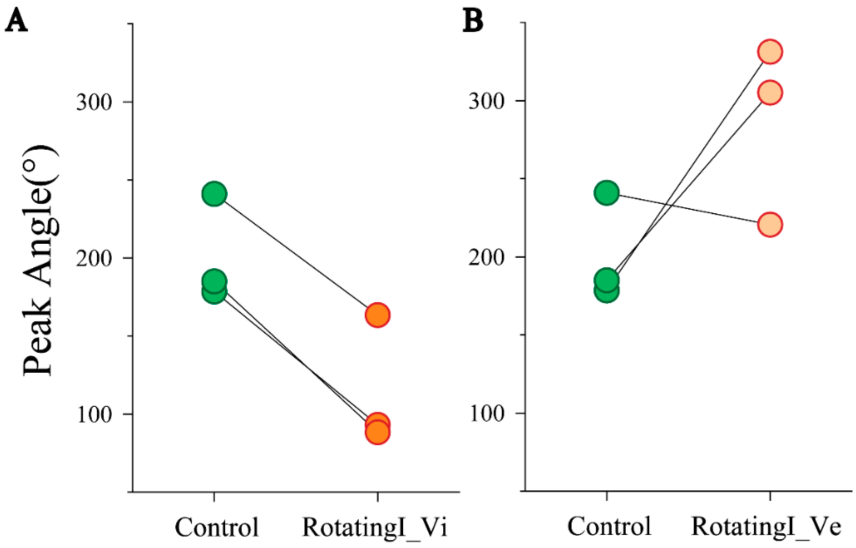

Figure S9. Changes of peak direction of three simultaneously recorded HD cells in the visual reference frame and vestibular reference frame from Control to RotatingI. (**A**) Changes of peak direction of HD cells under the visual reference frame showed that the shift of the peak direction is coherent. (**B**) Changes of peak direction of HD cells under the vestibular reference frame showed that the shift of the peak direction is scrambled.

Figure S10

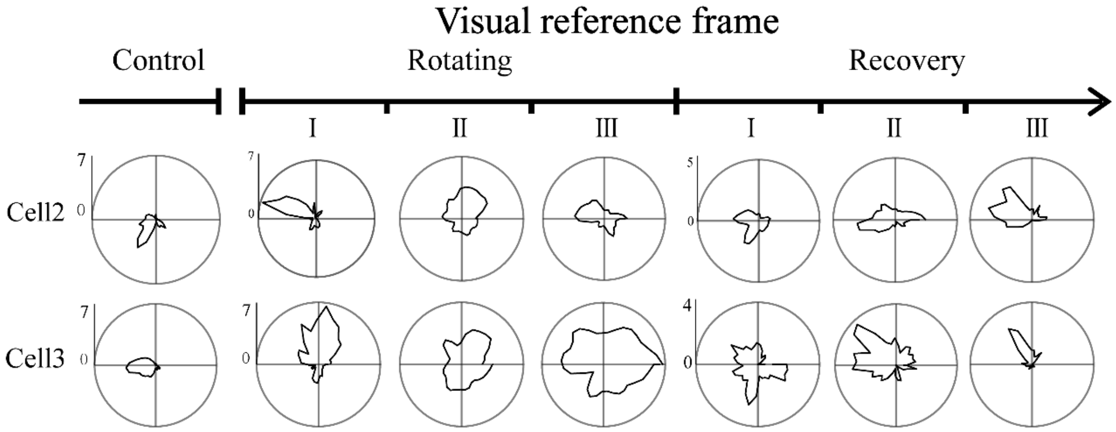

Figure S10. Detailed directional tuning curve changes of HD cells in the visual reference frame in Control, Rotating, and Recovery.

Figure S11

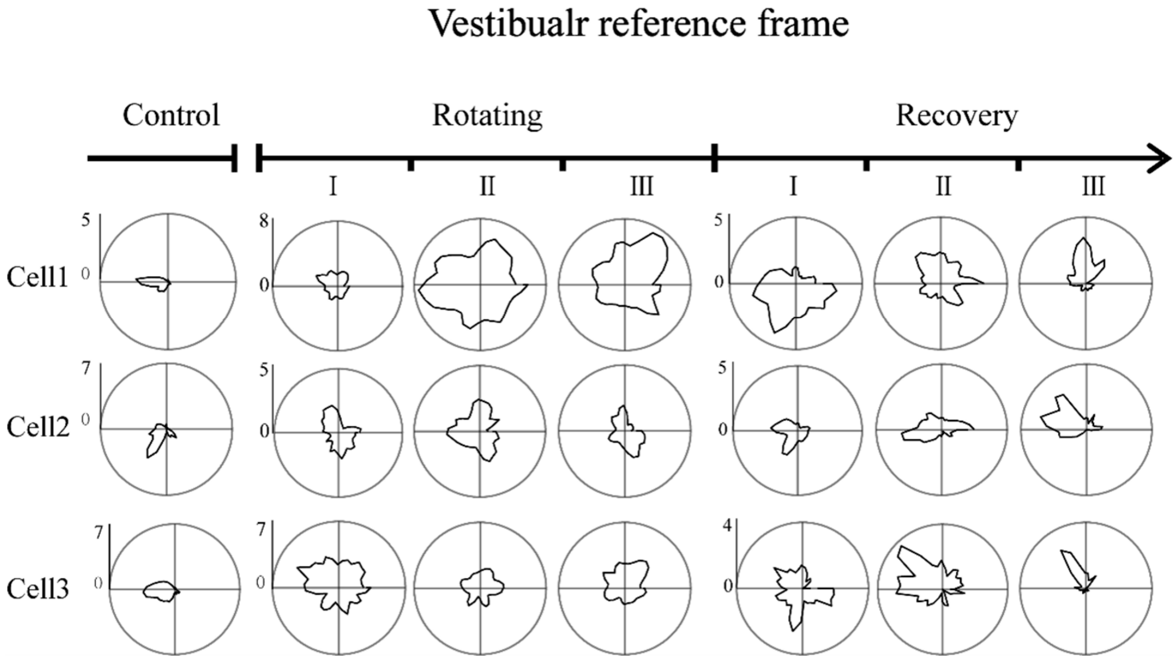

Figure S11. Detailed directional tuning curve changes of HD cells in the vestibular reference frame in Control, Rotating, and Recovery.

Figure S12

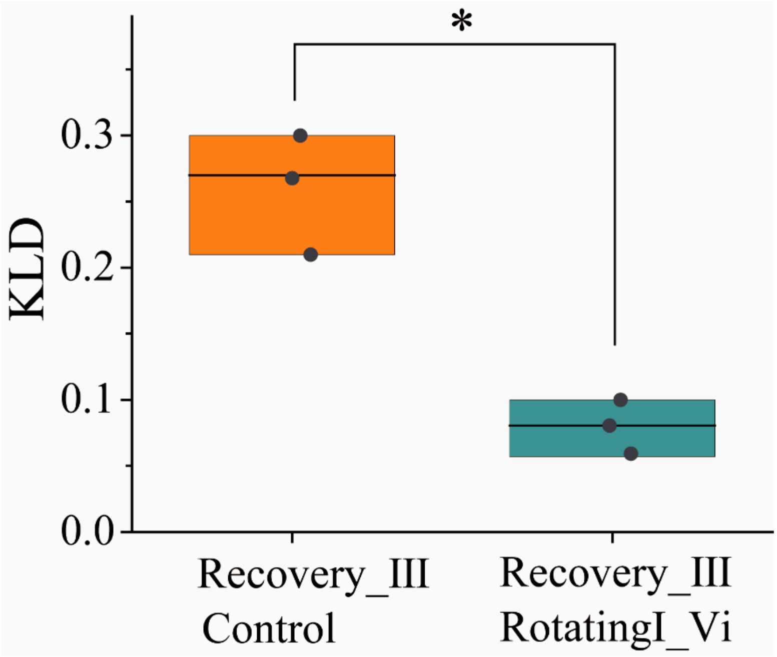

Figure S12. The divergence (KLD) of HD cells' tuning curves in RecoveryIII relative to RotatingI and Control revealed that the directional tuning of HD cells is closer to in RotatingI_Vi than in Control.

Figure S13

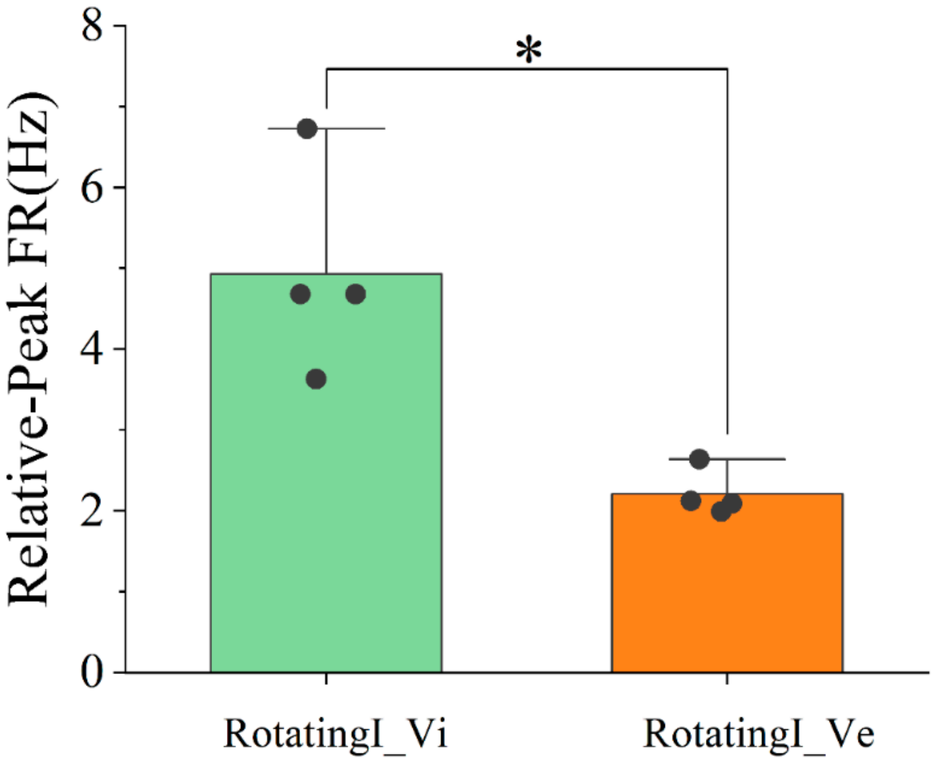

Figure S13. The Relative-Peak FR of RotatingI_Vi was significantly higher than that of RotatingI_Ve, indicating that HD cells showed more significant directional tuning in the visual reference frame than in the vestibular reference frame.

Figure S14

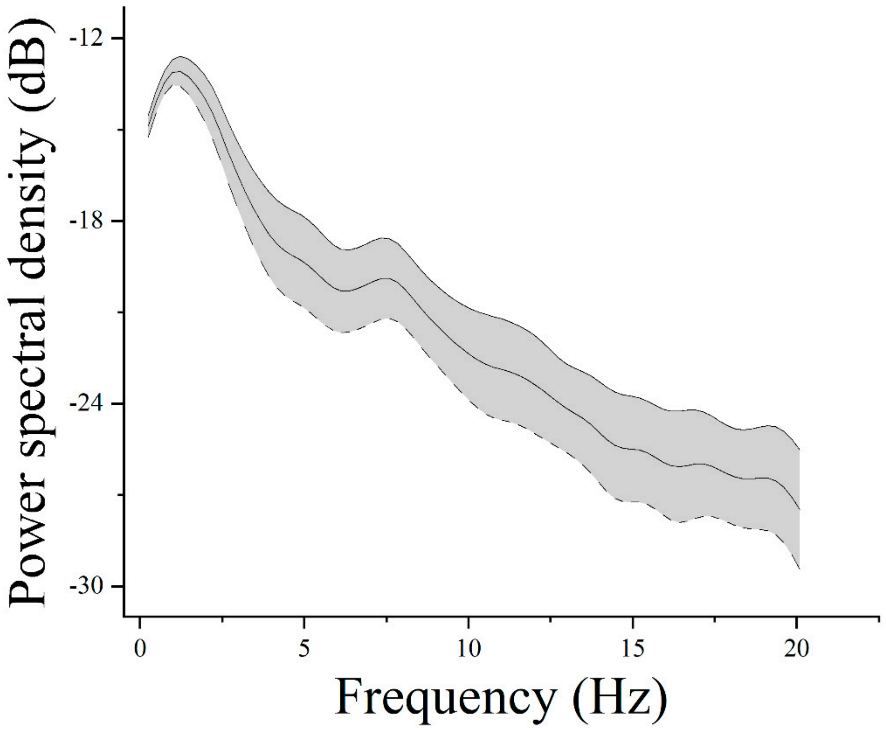

Figure S14. The PSD of LFP in RSC has a characteristic peak in the theta band.

Figure S15

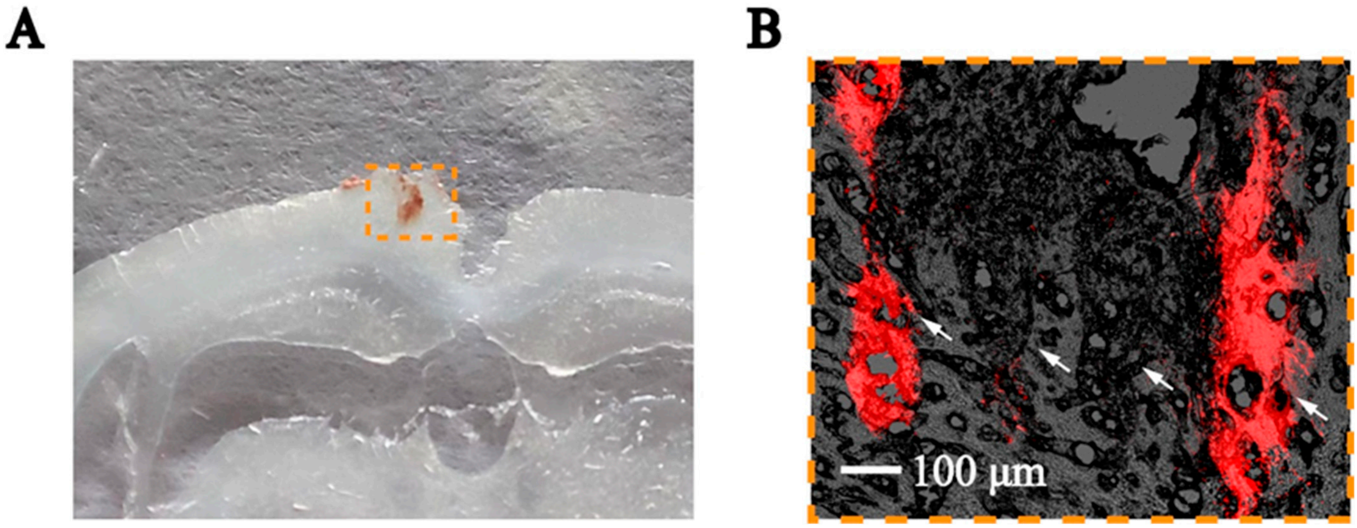

Figure S15. Postmortem histochemistry. (**A**) staining trajectory of electrode implantation in brain slices. (**B**) Dil fluorescence staining diagram of electrode implantation position.
